# Respiratory pathogen and clinical features of hospitalized patients in acute exacerbation of chronic obstructive pulmonary disease after COVID 19 pandemic

**DOI:** 10.1038/s41598-024-61360-4

**Published:** 2024-05-07

**Authors:** Soo Jung Kim, Taehee Kim, Hayoung Choi, Tae Rim Shin, Hwan Il Kim, Seung Hun Jang, Ji Young Hong, Chang Youl Lee, Soojie Chung, Jeong-Hee Choi, Yun Su Sim

**Affiliations:** 1 Division of Pulmonary, Allergy, and Critical Care Medicine, Department of Internal Medicine, Hallym University Kangnam Sacred Heart Hospital, Seoul, Korea; 2 Division of Pulmonary, Allergy, and Critical Care Medicine, Department of Internal Medicine, Hallym University Sacred Heart Hospital, Anyang-si, Korea; 3 Division of Pulmonary, Allergy, and Critical Care Medicine, Department of Internal Medicine, Hallym University Chuncheon Sacred Heart Hospital, Chuncheon-si, Korea; 4 Division of Pulmonary, Allergy, and Critical Care Medicine, Department of Internal Medicine, Hallym University Dongtan Sacred Heart Hospital, Dongtan-si, Korea; 5 Lung Research Institute, Hallym University College of Medicine, Chuncheon, Korea

**Keywords:** Respiratory pathogen, Chronic obstructive pulmonary disease, COVID-19, Chronic obstructive pulmonary disease, Infection

## Abstract

Respiratory infections are common causes of acute exacerbation of chronic obstructive lung disease (AECOPD). We explored whether the pathogens causing AECOPD and clinical features changed from before to after the coronavirus disease 2019 (COVID-19) outbreak. We reviewed the medical records of patients hospitalized with AECOPD at four university hospitals between January 2017 and December 2018 and between January 2021 and December. We evaluated 1180 patients with AECOPD for whom medication histories were available. After the outbreak, the number of patients hospitalized with AECOPD was almost 44% lower compared with before the outbreak. Patients hospitalized with AECOPD after the outbreak were younger (75 vs. 77 years, *p* = 0.003) and more often stayed at home (96.6% vs. 88.6%, *p* < 0.001) than patients of AECOPD before the outbreak. Hospital stay was longer after the outbreak than before the outbreak (10 vs. 8 days. *p* < 0.001). After the COVID-19 outbreak, the identification rates of *S. pneumoniae* (15.3 vs. 6.2%, *p* < 0.001) and *Hemophilus influenzae* (6.4 vs. 2.4%, *p* = 0.002) decreased, whereas the identification rates of *P. aeruginosa* (9.4 vs. 13.7%, *p* = 0.023), *Klebsiella pneumoniae* (5.3 vs. 9.8%, *p* = 0.004), and methicillin-resistant *Staphylococcus aureus* (1.0 vs. 2.8%, *p* = 0.023) increased. After the outbreak, the identification rate of influenza A decreased (10.4 vs. 1.0%, *p* = 0.023). After the outbreak, the number of patients hospitalized with AECOPD was lower and the identification rates of community-transmitted pathogens tended to decrease, whereas the rates of pathogens capable of chronic colonization tended to increase. During the period of large-scale viral outbreaks that require quarantine, patients with AECOPD might be given more consideration for treatment against strains that can colonize chronic respiratory disease rather than community acquired pathogens.

## Introduction

An acute exacerbation of COPD (AECOPD) is a key event in the natural history of the disease, associated with declining health, worsening lung function, and poor prognosis^1,2^.{, #9;(GOLD), 2023 #1} Such exacerbations can be triggered by various factors, among which respiratory infections are most common^3^. Bacteria including *Hemophilus influenzae*, *Moraxella catarrhalis*, *Streptococcus pneumoniae*, and *Pseudomonas aeruginosa*, as well as various viruses, are strongly associated with exacerbation.

The patterns of infection with respiratory viruses other than severe acute respiratory syndrome coronavirus 2 (SARS-CoV-2) changed during and after the global pandemic of coronavirus disease 2019 (COVID-19)^4^. The incidences of common, seasonal respiratory viral infections dramatically decreased. Influenza infections have been at historically low levels since 2020; the rates of infection by human metapneumovirus, enterovirus, adenovirus, respiratory syncytial virus (RSV), and human rhinovirus (HRV) have also substantially decreased^5^. Moreover, the rates of certain bacterial infections have fallen since the outbreak. An earlier prospective analysis of surveillance data showed that the transmission rates of *S. pneumoniae*, *H. influenzae*, and *Neisseria meningitidis* decreased in many countries worldwide; these changes were associated with significant reductions in life-threatening invasive diseases^6^.

Equally impressive decreases in COPD exacerbation rates were reported worldwide during and after the pandemic^7^, the declines ranged from 44 to 73% globally^7–10^. Such findings may be associated with reduction in transmission of respiratory pathogens causing AECOPD^3^. However, detailed data regarding changes in these pathogens after the COVID-19 outbreak have not been reported. Therefore, this study evaluated whether the pathogens causing AECOPD, and the clinical features of the condition, changed after the COVID-19 outbreak.

## Study design and methods

### Study design

This retrospective multicenter cohort study was performed in four hospitals within the Republic of Korea. We collected the medical records of patients with AECOPD admitted between January 2017 and December 2018 (before the outbreak of COVID-19) and between January 2021 and December 2022 (after the outbreak). The inclusion criteria were: a history of COPD diagnosed via post-bronchodilator forced expiratory volume in 1 s (FEV_1_)/forced vital capacity (FVC) < 0.7; admission with AECOPD; age > 40 years; and availability of all conventional test data for pathogens causing AECOPD.

AECOPD was defined by a need for additional medication or hospitalization because of worsening clinical symptoms such as cough, sputum production, and/or dyspnea based on the definition within the Global Initiative for Obstructive Lung Disease (GOLD) guidelines^1^.

### Variables

We collected demographic and clinicopathological information including age, sex, all comorbidities, lung function test results, smoking history, body mass index, exacerbation history, and medications used before AECOPD development. Clinical courses were evaluated in terms of intensive care unit admission and hospital mortality rates, inability to be discharged to home (i.e., transfer to a nursing hospital), and total hospitalization period.

Oral medications taken before AECOPD included xanthine derivatives, phosphodiesterase-4 inhibitors, and mucolytic agents. Inhaled treatments included long-acting beta 2-agonists (LABAs), long-acting muscarinic antagonists (LAMAs), inhaled corticosteroids, and combinations of these over ≥ 6 months before AECOPD development.

The microbiologic examination to identify the causative pathogen in patients with AECOPD was based on the first test performed upon hospitalization. Microbiological examinations included cultures of sputum or endotracheal aspirates; sputum polymerase chain reaction (PCR) tests for *Chlamydophila pneumoniae, H. influenzae, S. pneumoniae, Mycoplasma pneumoniae, Legionella pneumophila*, and viruses; serum tests for immunoglobulin M against *C. pneumoniae* and *M. pneumoniae*; urinary antigen tests for *L. pneumophila* and *S. pneumoniae*; and nasal swab tests for influenza A and B virus antigens. *C. pneumoniae* and *M. pneumoniae* were detected in respiratory specimens via PCR or (indirectly) in serum through immunoglobulin M measurements. *L. pneumophila* was detected in respiratory specimens via PCR and in urine using an antigen test. *H. influenzae* was detected in respiratory specimens by either PCR or culture. *S. pneumoniae* was detected in respiratory specimens by either PCR or culture, and in urine using an antigen test. All single or multiple pathogens identified were recorded.

### Statistical analysis

Absent pathogen test data were treated as missing values. Frequencies were expressed as numbers (%); descriptive data were expressed as medians with interquartile ranges. The chi-squared test or Fisher’s exact test was used to compare categorical variables; continuous variables were compared with the Mann–Whitney U test. Factors significantly associated with survival were subjected to Cox proportional hazards modeling after adjustment for age and tested by the log-rank test. The duration of in-hospital survival was defined as the time between admission and hospital discharge, as noted in medical records. Hazard ratios with 95% confidence intervals were calculated. The threshold for statistical significance was set to *p* < 0.05.

### Ethical approval

This study protocol was approved by the Institutional Review Board of Hallym University Kangnam (HKS 2023-11-008) Sacred Heart Hospital. All patient information was anonymized before analysis. Our institutional review boards (the Ethics Committee of Hallym University Kangnam Sacred Heart Hospital, Ethics Committee of Hallym University Sacred Heart Hospital, Ethics Committee of Hallym University Chuncheon Sacred Heart Hospital and the Ethics Committee of Ethics Committee of Hallym University Dontan Sacred Heart Hospital) approved this retrospective study and waived the requirement for informed consent from the patients. This study adhered to all relevant tenets of the 2013 revision of the Declaration of Helsinki.

## Results

### Patient characteristics

During the study period, 1186 patients with AECOPD were admitted to four hospitals. Among them, 418 patients were hospitalized after the outbreak of COVID-19, a decrease of approximately 44% over the same period compared to before the outbreak of COVID-19 (Table 1). The median age was 77 years, and 84% were men. After the COVID-19 outbreak, the patients were younger and more often men. In terms of comorbidities, congestive heart failure and liver cirrhosis were more common among patients admitted after the outbreak; the frequencies of other comorbidities did not differ between the two periods. The absolute FVC and FEV_1_ values were higher after the outbreak, but the predicted values did not differ, perhaps because the patients were younger compared with before the COVID-19 outbreak. Before COVID, more patients used LABAs or LAMAs alone than after the outbreak; LABA/LAMA combinations were more commonly utilized after the outbreak. Inhaled corticosteroid use did not differ between the two groups.

**Table 1:** Baseline demographic and clinical characteristics of patients with acute exacerbation of chronic obstructive pulmonary disease before and after outbreak of COVID 19.

	All patients (n = 1186)	Patients before COVID 19 (n = 713)	Patients after COVID 19 (n = 473)	*p*-value
Sex, male	997 (84%)	579 (82%)	418 (88%)	0.001
Age, years	77 (70–82)	77 (71–82)	75 (69–82)	0.003
BMI, Kg/m^2^	21.6 (19.1–24.3)	21.8 (19.2–24.3)	21.2 (19.0–24.2)	0.250
Pre-hospital				
Home	1089 (91.8%)	632 (88.6%)	457 (96.6%)	< 0.001
Health care center	14(1.2%)	11 (1.5%)	3 (0.6%)	0.156
Other hospital	83 (7.0%)	70 (9.8%)	13 (2.7%)	< 0.001
Exacerbation history	549 (46%)	340 (48%)	229 (44%)	0.237
Smoking history				
Current smoker	208 (22.7%)	113 (21.2%)	95 (21.2%)	0.201
Ex-smoker	494 (41.7%)	289 (53.8%)	205 (53.4%)	0.897
Pack-year	40 (25–50)	40 (20–50)	40 (30–50)	0.195
Co-morbidities				
Hypertension	535 (45.1%)	313 (43.9%)	222 (46.9%)	0.304
Diabetes mellitus	262 (22.1%)	149 (20.9%)	113 (23.9%)	0.224
Cancer	194 (16.4%)	98 (12.4%)	25 (8.3%)	0.059
Congestive heart disease	129 (11.0%)	87 (13.7%)	96 (20.3%)	0.003
Cerebrovascular disease	70 (5.9%)	45 (6.3%)	25 (5.3%)	0.463
Chronic kidney disease	62 (5.2%)	38 (5.3%)	24 (5.1%)	0.846
Liver cirrhosis	32 (2.7%)	10 (1.4%)	22 (4.7%)	0.001
Lung function				
FVC, L	2.46 (1.84–2.99)	2.431 (1.76–2.95)	2.47 (1.93–3.10)	0.019
FVC predicted, %	64 (53–76)	66 (53–75)	63 (52–77)	0.949
FEV_1_, L	1.11 (0.81–1.52)	1.06 (0.76–1.47)	1.16 (0.83–1.62)	0.01
FEV_1_ predicted, %	45 (33–59)	44 (32–58)	46 (33–61)	0.255
FEV_1_/FVC	50 (38–61)	48 (38–61)	51 (38–62)	0.135
Previous inhaler				
LABA	6 (0.5%)	6 (0.8%)	0	0.047
LAMA	987 (8.3%)	73 (10.3%)	25 (5.4%)	0.003
ICS/LABA	71 (6.0%)	45 (6.3%)	26 (5.6%)	0.604
LABA/LAMA	355 (29.9%)	176 (24.8%)	179 (38.5%)	< 0.001
ICS/LABA/LAMA	403 (34.0%)	251 (35.3%)	152 (32.7%)	0.356
Previous oral treatment				
PDE4 inhibitor	145 (12.2%)	93 (13.1%)	52 (11.2%)	0.329
Xanthine derivative	279 (23.5%)	156 (21.9%)	123 (26.5%)	0.075
Mucolytic agent	482 (40.6%)	288 (40.5%)	194 (41.7%)	0.679
Admission				
Intensive care unit	182 (15.3%)	107 (15.0%)	75 (15.9%)	0.687
Outcome				
Hospital days	9 (6–14)	8 (5–12)	10 (6–17)	< 0.001
Death	70 (5.9%)	40 (5.6%)	30 (6.3%)	0.604
Discharge to other hospital	58 (4.9%)	45 (6.4%)	13 (2.7%)	0.005

Values are presented as number (%) or median value (interquartile range). BMI, body mass index; FVC, forced vital capacity; FEV1, forced expiratory volume in one second; LABA, long-acting beta 2-agonist; LAMA, long-acting muscarinic antagonist; ICS, inhaled corticosteroid; PDE4, Phosphodiesterase-4.

After COVID-19, the number of patients coming from home has increased, and the number of patients discharged to other hospitals after treatment has also decreased. Although the patient’s hospitalization period became longer after COVID-19 outbreak, there was no difference in the intensive care unit hospitalization rate or mortality rate between the two groups.

### Microbiological analysis: changes after the COVID-19 outbreak

**Figure 1:**
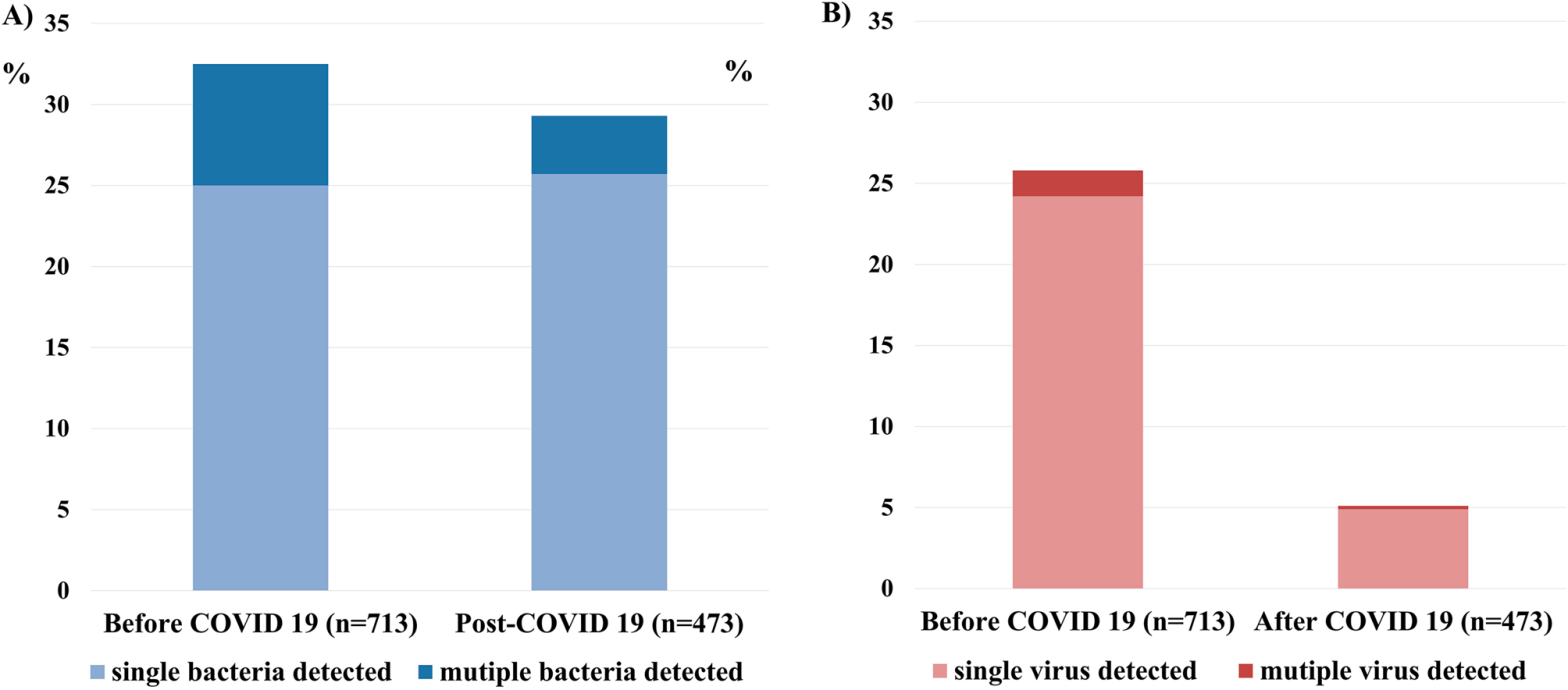
Overall bacterial and viral detection rates during AECOPD before and after the COVID-19 outbreak: (**A**) Bacteria. (**B**) Viruses.

**Figure 2:**
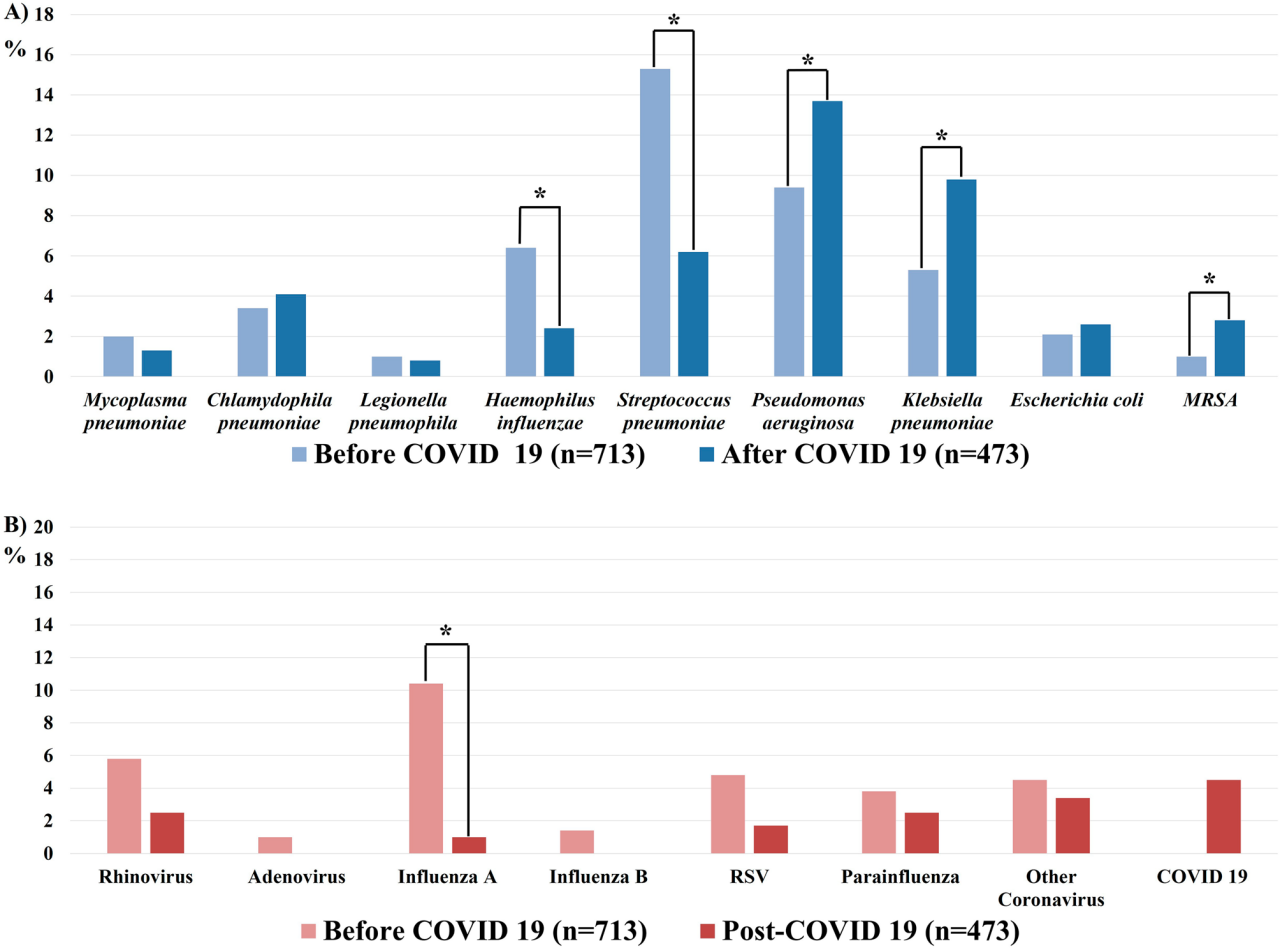
Bacterial and viral detection rates during AECOPD before and after the COVID-19 outbreak: (**A**) Detection rates of all bacterial species. (**B**) Detection rates of all viral species. *p < 0.05. MRSA, methicillin-resistant *Staphylococcus aureus.* RSV, respiratory syncytial virus. COVID-19, coronavirus disease 2019.

Detection rate of bacteria and virus after COVID-19 outbreak were 29.3% and 5.1%, respectively, which has decreased compared over same period before COVID-19 outbreak (Fig. 1). Before the outbreak, the most frequently identified bacteria were *S. pneumoniae* (11.7%) and *P. aeruginosa* (11.1%). However, after the outbreak, the identification rates of *S. pneumoniae* (15.3 vs. 6.2%, *p* < 0.001) and Hemophilus influenzae (6.4 vs. 2.4%, *p* = 0.002) decreased, whereas the identification rates of *P. aeruginosa* (9.4 vs. 13.7%, *p* = 0.023), *K.pneumoniae* (5.3 vs. 9.8%, *p* = 0.004), and methicillin-resistant *Staphylococcus aureus* (MRSA) (1.0 vs. 2.8%, *p* = 0.023) increased. The detection rates of all viruses except COVID-19 considerably decreased after the outbreak. In particular, the influenza A detection rate decreased from 10.4% to 1% (Fig. 2, Table 2). There were 44 cases (5%) in total with mixed bacterial and viral infections, 41 cases (6.6%) before the COVID-19 outbreak and 3 cases (0.6%) after the COVID-19 outbreak (Table 2).

**Table 2:** Analysis of bacterial and viral species in patients with acute exacerbation of chronic obstructive pulmonary disease before and after outbreak of COVID 19.

	All patients (n = 1186)	Patients before COVID 19 (n = 713)	Patients after COVID 19 (n = 473)	*p*-value
Bacterial class				
Single bacteria detected	25.3 (297/1186)	25.0 (177/713)	25.7 (120/473)	0.799
Multiple bacterial detected	6.0 (70/1186)	7.5 (53/713)	3.6 (17/473)	0.006
Bacteria				
*Mycoplasma pneumoniae*	1.8 (15/855)	2.0 (10/512)	1.5(5/343)	0.589
*Chlamydophila pneumoniae*	3.7 (31/847)	3.4 (17/503)	4.1 (14/344)	0.599
*Legionella pneumophila*	0.9 (7/790)	1.0 (4/407)	0.8 (3/383)	0.765
*Hemophilus influenzae*	4.8 (55/1153)	6.4 (44/690)	2.4 (11/436)	0.002
*Streptococcus pneumoniae*	11.7 (136/1165)	15.3 (107/699)	6.2 (29/466)	< 0.001
*Moraxella catarrhalis*	0.3 (4/1143)	0.9 (4/683)	0	0.104
*Pseudomonas aeruginosa*	11.1 (127/1143)	9.4 (64/683)	13.7 (63/460)	0.023
*Klebsiella pneumoniae*	7.1 (81/1143)	5.3 (36/683)	9.8 (45/460)	0.004
*Escherichia coli*	2.3 (26/1143)	2.1 (14/683)	2.6 (12/460)	0.539
MSSA	0.5 (6/1143)	0.3 (2/683)	0.9 (4/460)	0.186
MRSA	1.7 (20/1143)	1.0 (7/683)	2.8 (13/460)	0.023
*Stenotrophomonas maltophilia*	0.5 (6/1143)	0.6 (4/683)	0.4 (2/460)	0.615
Others	4.8 (56/1162)	4.3 (30/696)	5.6 (26/466)	0.322
Virus class				
Sigle virus detected	14.1 (125/1186)	24.2 (102/713)	4.9 (23/473)	< 0.001
Multiple virus detected	0.9 (9/1186)	1.1 (8/713)	0.2 (1/473)	0.071
Virus				
Rhinovirus	4.9 (20/410)	5.8 (17/292)	2.5 (3/118)	0.163
Adenovirus	0.7 (3/411)	1.0 (3/293)	0	0.270
Influenza A	8.9 (47/529)	10.4 (43/415)	3.5 (4/114)	0.023
Influenza B	1.9 (10/526)	1.4 (9/412)	1 (1/114)	0.366
RSV	3.9 (16/411)	4.8(14/293)	1.7 (2/118)	0.144
Parainfluenza	3.4 (14/411)	3.8 (11/293)	2.5 (3/118)	0.531
Other Coronavirus	4.2 (17/408)	4.5 (13/290)	3.4(4/118)	0.616
COVID-19	4.5 (21/464)	0	4.5 (21/464)	0.384

Values are presented as percent (%). Parentheses are indicated as (number of detected pathogens/number of performed tests). MSSA, methicillin-sensitive *Staphylococcus aureus*; MRSA, methicillin-resistant *Staphylococcus aureus*; RSV, respiratory syncytial virus; COVID-19, coronavirus disease 2019.

### Factors prognostic of mortality

Before the outbreak, histories of admission to another hospital and/or COPD exacerbation, and the presence of *K. pneumoniae* and/or MRSA, were prognostic of mortality. After the outbreak, only MRSA detection was prognostic of mortality (Table 3).

**Table 3:** Prognostic factor for mortality of hospitalized patents in acute exacerbation of chronic obstructive pulmonary disease before and after outbreak of COVID 19 adjusted for age.

Characteristics	Before COVID 19			After COVID 19		
	HR	CI	*p*-value	HR	CI	*p*-value
Other hospital before admission	2.257	1.037–4.915	0.040	4.202	1.272–13.884	0.019
History of Exacerbation	2.394	1.235–4.640	0.010	1.667	0.805–3.414	0.171
Diabetes mellitus	0.411	0.146–1.155	0.092	0.636	0.244–1.663	0.356
Single bacteria detected	2.228	1.190–4.171	0.012	1.319	0.601–2.897	0.490
Multiple bacteria detected	1.030	0.995–1.065	0.095	4.287	1.496–12.290	0.007
*Mycoplasma pneumoniae*	1.952	0.265–14.401	0.512			
*Hemophilus influenzae*	0.816	0.197–3.388	0.780	3.631	0.851–15.494	0.082
*Streptococcus pneumoniae*	1.155	0.511–2.611	0.729	0.469	0.064–3.457	0.458
*Pseudomonas aeruginosa*	1.586	0.617–4.079	0.338	0.702	0.213–2.319	0.562
*Klebsiella pneumoniae*	2.636	1.030–6.749	0.043	1.553	0.539–4.477	0.415
*Escherichia coli*	1.151	0.157–8.422	0.890	2.939	0.695–12.425	0.143
MRSA	5.627	1.355–23.368	0.017	3.856	1.164–12.773	0.027
Sigle virus detected	0.950	0.377–2.393	0.913	0.628	0.148–2.675	0.530
Influenza A	0.724	0.170–3.071	0.661			
Influenza B	4.405	1.038–18.698	0.044			
Parainfluenza	1.735	0.229–13.139	0.594	5.615	0.692–45.591	0.106
Other Coronavirus	1.661	0.212–12.988	0.629			
COVID-19				0.688	0.094–5.058	0.713

HR, hazard ratio; CI, confidence interval.

## Discussion

The present study revealed changes in the AECOPD hospitalization rates and isolation rates of corresponding respiratory pathogens after the COVID-19 outbreak. The number of patients hospitalized with AECOPD decreased by approximately 44% compared with the number over the same period before the outbreak. Similar phenomena have been reported globally. In the United States, an analysis of the Veterans Affairs Corporate Data Warehouse, a national repository of electronic health records created during visits to all Veterans Affairs facilities, revealed a 48.4% decline in COPD admissions to Veterans Affairs hospitals after the outbreak^8^. In the United Kingdom, analysis of data from Public Health Scotland and the Secure Anonymized Information Linkage Databank of Wales revealed a 48% pooled reduction in AECOPD requiring hospital admission^9^. In Singapore, the monthly rate of acute COPD admissions decreased by more than 50% in the first 5 months (February–July 2020) after the outbreak^10^.

Decreases in AECOPD may be associated with reduced transmission of respiratory-associated pathogens. Most COPD exacerbations are caused by bacterial or viral infections^3^. Globally, the incidence of AECOPD and transmission levels of respiratory viruses simultaneously decreased^11^. The present study also showed that the overall detection rates of bacteria and viruses decreased after the outbreak, explaining the observed AECOPD reduction.

*H. influenzae*, *M. catarrhalis*, *S. pneumoniae*, and *P. aeruginosa* were commonly isolated from AECOPD patients in previous studies^12^. The most commonly detected viruses in such patients were rhinovirus, influenza A, and RSV^13^. As in a previous study^12,13^, *S. pneumonia* and *P. aeruginosa* were the frequently identified bacteria, whereas influenza A and rhinovirus were the most common viruses, in the present study. *H. influenzae*, *M. catarrhalis*, and RSV were also detected in the present work.

Notably, we found that pathogen detection rates changed after the COVID-19 outbreak. The incidences of *S. pneumoniae*, *H. influenzae,* and all viruses except COVID-19 significantly decreased after the outbreak. Such changes have been reported worldwide. A previous study demonstrated significant and sustained reductions in invasive diseases caused by *S. pneumoniae*, *H. influenzae*, and *N. meningitidis,* beginning in early 2020^6^. All viral detection rates have declined worldwide. The Centers for Disease Control and Prevention reported a 98% decrease in influenza activity, from a median of 19.34% to 0.33%, in the United States^14^. Southern Hemisphere countries (Australia, Chile, and South Africa) have also reported minimal influenza activity. Furthermore, the detection rates of RSV, rhinovirus, metapneumovirus, and parainfluenza virus have decreased^4^. Consistent with previous international reports, we found that the influenza detection rate substantially decreased from 10.4 to 1.0%.

The changes may be partly explained by the widespread introduction of COVID-19 lockdown policies. *S. pneumoniae* and *H. influenzae* are typically transmitted person-to-person via the respiratory route^15^. Respiratory viruses are transmitted in respiratory droplets and aerosols^16^. Therefore, widespread adoption of COVID-19 containment policies, such as social distancing and the use of face masks in public spaces, may have reduced the transmission rates of respiratory-related pathogens and COVID-19. The COVID-19 containment policies and relevant public information campaigns slowed the transmission of respiratory-related pathogens, thereby reducing AECOPD rates. Therefore, COVID-19 containment policies are effective in lowering AECOPD levels. Although long-term implementation of strict COVID-19-like containment policies is impossible considering the socioeconomic costs, the COVID-19 experience may aid the establishment of strategies to prevent AECOPD.

The *S. pneumoniae* and respiratory virus detection rates significantly declined, but the detection rates of *P. aeruginosa* and MRSA significantly increased, after the COVID-19 outbreak. Both *P. aeruginosa* and MRSA are associated with poor AECOPD outcomes^17,18^. In other studies, *K.pneumoniae, P.aerusinosa*, and MRSA were reported as strains causing colonization in COPD patients^19–22^. Additionally, approximately 20% of hospitalized COPD patients is colonized with MRSA^18^. These pathogens have the capacity to engage in chronic colonization; the corresponding numbers may be less affected by COVID-19 quarantine policies, compared with the numbers of other pathogens. Colonization precedes obvious clinical infection. Murphy et al.^23^ reported that exacerbations caused by *P. aeruginosa* were more common in patients with advanced COPD compared with early COPD. The median predicted FEV_1_ of patients in the present study was 45% and almost half of the patients had a history of AECOPD, reflecting the severity of COPD. These results may explain the increased detection rate of *P. aeruginosa* after the COVID-19 outbreak.

MRSA is associated with adverse outcomes among patients with AECOPD. Narewski et al.^18^ reported that COPD patients colonized with MRSA had longer hospitalizations, required longer courses of antibiotics, and was more likely to require intensive care. Additionally, persistent infection with MRSA in patients with cystic fibrosis was associated with a more rapid rate of decline in lung function^24^. Similarly, we found that MRSA infection was prognostic of mortality before and after the COVID-19 outbreak. COPD patients colonized with MRSA may require close attention.

The present work was a multicenter study including a large number of patients and we comprehensively evaluated the changes in pathogens infecting AECOPD patients before and after the COVID-19 outbreak. However, our study had some limitations. First, this was a retrospective work. Second, we could not evaluate the pathogen status of AECOPD patients after COVID-19 containment policies were completely lifted. It remains unclear whether the observed changes have persisted since easing began. In May 2023, the World Health Organization declared that the COVID-19 public health emergency was over. Thus, an additional study is needed. Third, changes in healthcare utilization by patients with non-COVID-19 conditions during and after the outbreak may have affected pathogen detection rates^25–27^. Forth, this study was conducted on patients hospitalized for acute exacerbations of COPD, patients with severe bronchiectasis were not included, but there may have been patients with focal bronchiectasis, and there is a lack of distinction between respiratory structural abnormalities and their impact on microbial colonization. In the present study, the numbers of patients transferred from other hospitals and discharged to other hospitals after treatment both decreased during and after the outbreak. These findings may be related to difficult transfer between hospitals during lockdown, which could have impacted the pathogen detection rates.

## Conclusion

After the COVID-19 outbreak, the number of hospitalized AECOPD patients decreased by almost 44% compared with the number during the same period before the pandemic. After the outbreak, the incidences of community-transmitted AECOPD pathogens tended to decrease, whereas the incidences of pathogens capable of chronic colonization tended to increase. The widespread introduction of COVID-19 containment policies, such as social distancing, may have lowered the transmission of respiratory-associated pathogens, thereby lowering the incidence of AECOPD. When MRSA was identified in AECOPD, patients had a high mortality rate both before and after the COVID-19 outbreak. Regardless of the viral outbreak situation, it is very important to consider treatment for strains associated with chronic colonization or drug resistance in AECOPD patients. However, since strains related to chronic colonization are detected at a higher frequency in AECOPD patients in large-scale viral outbreak situation, chronic colonization strains might need to be given more consideration in the treatment of AECOPD patients in outbreak.

## Data Availability

The datasets used and analyzed during the present study are available from the corresponding author on reasonable request.
